# Surgical Reconstruction of Elbow Extension in Spinal Cord Injury and Tetraplegia: A Systematic Review

**DOI:** 10.1016/j.jhsg.2023.11.012

**Published:** 2024-01-02

**Authors:** Marvin Man Ting Chung, Gabriel Ching Ngai Leung, Wing Yuk Ip

**Affiliations:** ∗ Department of Orthopaedics & Traumatology, University of Hong Kong, Hong Kong SAR; † Department of Orthopaedics & Traumatology, Queen Mary Hospital, Hong Kong SAR

**Keywords:** Elbow extension, Nerve transfer, Spinal cord injury, Tendon transfer, Tetraplegia

## Abstract

**Purpose:**

Surgical reconstruction of elbow extension can help restore function in patients with tetraplegia and triceps paralysis because of spinal cord injury. Both posterior deltoid-to-triceps tendon transfer and transfer of the branch of the axillary nerve to the triceps motor branch of the radial nerve have been described for triceps reanimation. This systematic review aimed at reviewing current evidence in the two schools of surgery in terms of their outcome and complication profile.

**Methods:**

A systematic review was conducted using MEDLINE (1974–2023) and EMBASE (1946–2023) databases. The keyword terms “elbow extension,” “triceps,” “deltoid,” “nerve transfer,” “spinal cord injury,” “tetraplegia,” “quadriplegia,” and “tetraplegic” were used in the initial search, which was supplemented with manual searches of the bibliographies of retrieved articles.

**Results:**

Twenty studies met our inclusion criteria, with 14 studies (229 limbs) on posterior deltoid-to-triceps tendon transfer, 5 studies (23 limbs) on axillary to radial nerve transfer, and 1 study (1 limb) on combined transfer. For the tendon transfer group, the majority of studies reported a median triceps power of grade 3, with a wide range of failure percentage to reach antigravity (0% to 87.5%). Common complications included gradual stretching of the musculotendinous unit, rupture of the tendon transferred, elbow contracture, and infection. For the nerve transfer group, the majority of studies also reported a median triceps power reaching grade 3. There were no reported complications or loss of power in donor action of shoulder abduction or external rotation.

**Conclusions:**

Transfer of the axillary nerve branch to the triceps motor branch of the radial nerve in tetraplegia shows promising results, with comparable triceps muscle power compared to traditional tendon transfer and a low incidence of complication.

**Type of Study/Level of Evidence:**

Systematic Review III.

Patients with tetraplegia as a result from cervical spinal cord injury suffer from significant morbidities. After the acute recovery and subsequent rehabilitation process, they often prefer further improvements in upper limb function compared to other functions.^1^ As midcervical spine injury is more common, many of them lack adequate innervation of C7 muscle groups, resulting in weak elbow extension. Surgical reconstruction of elbow extension in patients with spinal cord injury allows improvement in activities of daily living, transfer, overhead activities, and pressure sore prevention.^2^ Different surgical methodologies exist in the literature on surgical reconstruction of elbow extension with various techniques and modifications. Traditionally, tendon transfer was the mainstay of treatment in reconstructing elbow extension, using the posterior deltoid or biceps as the donor tendon for transfer to the triceps tendon. Tendon transfer also requires prolonged immobilization for protection postoperatively with alternation in biomechanics.^3^ On the other hand, nerve transfer has been gaining popularity among hand surgeons over the past decade, with a paradigm shift not only in spinal cord injury but also in various paralytic conditions of the upper limb. Nerve transfer from the posterior branch of the axillary nerve to the long head of the triceps branch of the radial nerve has been described with low donor morbidity and reasonable outcome, but this technique comes with the inherent disadvantages of nerve transfer, such as less predictability and the long period required before recipient motion is seen.^4^^,^^5^

At present, there exists no concrete evidence and systematic review demonstrating the gold standard in such surgical reconstruction of elbow extension in patients with tetraplegia. Although there was a previous systematic review of posterior deltoid-to-triceps tendon transfer in 2009 by Hamou et al^6^, nerve transfers have been gaining favor among hand surgeons with a surge in nerve transfer techniques, thus rendering an updated review of techniques of elbow extension surgical reconstruction, in particular a comparison of nerve transfer and tendon transfer, necessary. We therefore conducted a systematic review aimed at reviewing the current evidence on surgical reconstruction of elbow extension in the context of tetraplegia and spinal cord injury, comparing deltoid-to-triceps tendon transfer with the transfer of the posterior branch of the axillary nerve to the long head of the triceps branch of the radial nerve in terms of functional outcome and complication profiles. The posterior deltoid tendon transfer was specifically chosen for direct comparison with the axillary nerve to radial nerve branch transfer because they both use and sacrifice similar groups of muscles as a synergistic transfer, which have no co-contraction issues as noted in the case of an antagonistic transfer, such as biceps-to-triceps transfer.^7^

## Materials and Methods

A query of studies written in English and available on MEDLINE from 1974 to March 2023 and Embase from 1946 to March 2023 was performed using Ovid database. Preferred Reporting Items for Systematic Reviews and Meta-Analyses guidelines were used for the article search and reporting of the systematic review. The keyword terms “elbow extension,” “triceps,” “spinal cord injury,” “tetraplegia,” “quadriplegia,” and “tetraplegic” were used in the initial search. The search was further supplemented with manual searches of bibliographies of retrieved articles. Inclusion criteria included studies reporting the outcome of surgical treatment in the reconstruction of elbow extension, including tendon and nerve transfer. Case reports were also included in the review to obtain more studies and patients because surgical reconstruction of elbow extension is not a widely performed procedure and new modifications are constantly evolving. Exclusion criteria included non-English articles; technical descriptions of procedures without report of outcomes; nonsurgical reconstructions of elbow extension, such as functional electrical stimulation; reconstruction of elbow extension in patients with brachial plexus injury or isolated peripheral nerve palsy; editorials; and duplicates. Articles first underwent initial review of titles and abstracts where articles were rejected based on exclusion criteria. After the initial review, potential articles were further assessed for possible inclusion in detail. Final analysis was then performed based on the final number of articles included. Two orthopedic surgeon reviewers independently reviewed the articles, and differences between the reviewers’ opinions were resolved through consensus. Data retrieved included number of patients and limbs, mean age, type of surgery performed, assessment of preoperative and postoperative elbow extension muscle strength based on Medical Research Council (MRC) grading, preoperative and postoperative donor muscle strength based on MRC grading, follow-up duration, and the rate and type of complications.

**Figure 1:**
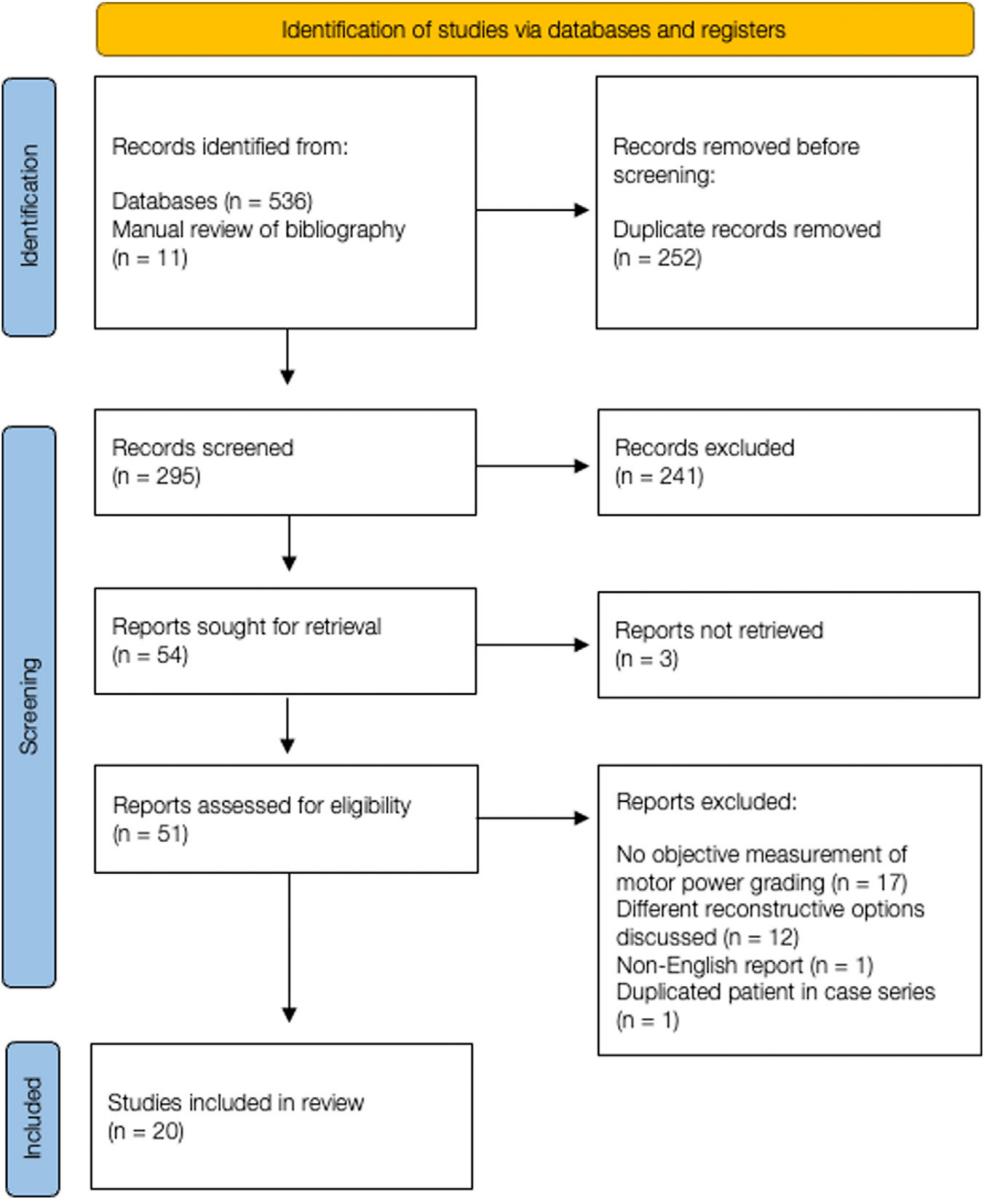
Flow diagram of article selection process.

The initial literature search yielded 547 articles. In total, 252 articles were removed due to duplicate records, 241 were excluded after initial screening of titles and abstracts, and 3 were excluded as the reports were unable to be retrieved. After further detailed assessment for possible inclusion and exclusion criteria, 31 articles were further excluded for various reasons charted in the flow diagram of article selection process (Fig. 1). Twenty articles met the final inclusion criteria and were included in the systematic review.

## Results

Among the 20 studies that met the final inclusion criteria, there were 14 studies with 229 limbs subject to posterior deltoid-to-triceps tendon transfer, 5 studies with 23 limbs subject to transfer of the axillary nerve branch to the triceps motor branch of the radial nerve, and 1 study with 1 limb subject to a combined tendon and nerve transfer.

For the tendon transfer group, the preoperative median MRC power of triceps ranged from 0 to 1. The mean age ranged from 22.4 to 37 years. In total, 12 out of 14 studies reported a median postoperative MRC power of triceps of greater than 3 in the tendon transfer group. The proportion of patients who failed to reach antigravity triceps motion (ie, MRC grading less than 3) varied among different cohorts and was reported to range from 0% to 87.5%. Complications were also reported widely, with the most common ones being gradual stretching of the musculotendinous unit, rupture of the tendon transfer or anastomosis site, and infection (Table 1).^8^, ^9^, ^10^, ^11^, ^12^, ^13^, ^14^, ^15^, ^16^, ^17^, ^18^, ^19^, ^20^, ^21^

**Table 1:** Studies Included in the Tendon Transfer Group

Study	Year	Number of Patients	Number of Limbs	Mean Age (Y)	Median Triceps MRC Power Pretransfer (Range)	Median Triceps MRC Power Post-transfer (Range)	Failure to Reach Antigravity (MRC < 3)	% Failure to Reach Antigravity	Follow-Up Duration (Months)	Complications
Bryan^11^	1977	7	14	22.4	NR	3.5 (2–4)	1	7.1	NR	1 slipped tendon insertion 1 olecranon bursa infection
Debenedetti^9^	1979	13	14	NR	0.5 (0–2)	3.6∗	NR	NR	13.8 (6.5–18.5)	1 infection 2 stretching of muscle tendon unit
Lamb and Chan^12^	1983	10	16	NR	NR	4.5 (3-5)	0	0	NR	1 flexion contracture and loss of extension power
Raczka et al^13^	1984	18	19	NR	0 (0–2)	3.5 (0–4.5)	6	31.6	49 (7–79)	1 heterotopic ossification at deltoid insertion and posterior deltoid 1 tendon graft failure/attenuation 2 deep wound infection 1 progressive bilateral deltoid decline due to C5 root injury
Lacey et al^14^	1986	10	17	NR	0	3 (2–4)	1	5.9	27.1 (6–48)	1 rupture after forced manipulation 1 stitch abscess
Johnstone et al^15^	1987	7	8	30.6	0	3 (2–4)	3	37.5	27.6 (12–41)	1 adhesion and elongation at the point of reflection of the triceps tendon despite tenolysis
Vanden Berghe et al^16^	1991	6	8	26.7	NR	3 (3–4)	0	0	NR	1 rupture of transfer after fall
Mohammed et al^17^	1992	NR	24	NR	0	3 (0–4)	7	29.2	32	1 rupture anastomosis
Paul et al^18^	1994	9	10	29	0 (0–2)	3.5 (2–4)	2	20	31 (20–42)	NR
Welraeds et al^19^	2003	10	12	37	0	2 (0–4)	3	25	4	1 suture slackening requiring re-do 1 transfer slackening requiring re-tension
Mulcahey et al^10^	2003	7	8	NR	NR	2 (1–3)	7	87.5	24	NR
Turcsanyi et al^20^	2010	10	15	26	1 (1–2)	4 (3–5)	0	0	10 (5–19)	1 wound hematoma 1 loss of elbow flexion strength requiring Z-tenotomy
Wangdell et al^8^	2012	14	19	30	0 (0–1)	4 (2–5)	2	10.5	12	None
Carre et al^21^	2022	36	45	30	0	3.7∗	NR	NR	23 (13–39)	12 transfer relaxation/ intolerance to synthetic Dacron ligament

MRC, Medical Research Council grading; NR, not reported.

∗ Only average/mean data reported in the series without range or raw data included.

In the nerve transfer group, the mean age ranged from 19 to 36 years. In total, 4 out of 5 studies reported a median postoperative MRC power of triceps of greater than 3 at a mean follow-up of 20.2 months. None of the patients failed to reach antigravity triceps motion. Notably, there was also no reported loss of power in donor action of shoulder abduction (from the posterior, middle, or anterior deltoid branch of the axillary nerve) or external rotation (from the teres minor branch of the axillary nerve). There was also no report of complications or infection among the studies in the nerve transfer group (Table 2).^4^^,^^22^, ^23^, ^24^, ^25^, ^26^

**Table 2:** Studies Included in the Nerve Transfer Group

Study	Year	Number of Patients	Number of Limbs	Mean Age	Donor Nerve	Recipient Nerve	Approach	Median Triceps MRC Power Pretransfer (range)	Median Triceps MRC Power Post-transfer (Range)	Median Shoulder MRC Power Pretransfer (Range)	Median Shoulder MRC Power Post-transfer (Range)	Failure to Reach Antigravity (MRC < 3)	% Failure to Reach Antigravity	Follow-Up Duration (Months)	Complications
Nehete et al^26^	2020	2	4	19	2: Anterior division of axillary nerve 1: Branch of middle deltoid 1: Posterior division of axillary nerve	Long and upper medial head branch	Axillary	NR	3 (0–4)	NR	NR	2	50	30 (24–36)	NR
Bertelli and Ghizoni^4^	2015	7	13	26.6	9: Posterior deltoid branch 2: Posterior + middle deltoid branch 2: Anterior deltoid branch	Long and upper medial head branch	Axillary	NR	4 (3–4)	3.6 (Abd)	3.9 (Abd)	0	0	20.1 (17–24)	None
Bertelli et al^23^	2011	1	2	21	TM branch	Long head branch	Axillary	0	4 (4-4)	5 (ER)	5 (ER)	0	0	14	None
Fox et al^24^	2015	2	3	36	Posterior deltoid branch	Long / medial triceps branch	Posterior	0	2.5 (2.5)	NR	NR	NR	NR	12	NR
van Zyl et al^25^	2014	1	1	21	TM branch	Long head branch	Axillary	0	4	5 (ER)	5 (ER)	0	0	19	None
Biondi et al^22^ (combined)	2020	1	1	21	TM branch	Long head branch	Posterior (together with tendon transfer)	2	4	5 (ER)	5 (ER)	0	0	17	None

Abd, abduction; ER, external rotation; MRC, Medical Research Council grading; TM, teres minor.

There was also one case report describing a combined approach using both a posterior deltoid-to-triceps tendon transfer and transfer of the teres minor branch of the axillary nerve to the long head of the triceps motor branch of the radial nerve, as noted in Table 2.^22^ Postoperative power reached MRC grade 4 at 17 months, and, again, no complications or loss of donor strength was reported.

## Discussion

This systematic review showed that both posterior deltoid-to-triceps transfer and transfer of the axillary nerve branch to the triceps motor branch provide comparable triceps muscle power in terms of MRC grading. Quantitative measurements were not available in the majority of studies. The rate of successfully reconstructing antigravity triceps power in the tendon transfer group was quite variable, possibly signifying a heterogeneous patient group and variations in surgical techniques and surgeon factors. As the goal of reconstructive upper limb surgery was to reach meaningful and functional muscle power, an MRC power less than grade 3, or failure to reach antigravity strength, was classified as a suboptimal outcome. Nevertheless, Koch-Borner et al^27^ reported that even when there was subgravity improvement in muscle strength, patients could still gain functional improvement and some active motion that assisted their activities of daily living, and their activity performance score and patient satisfaction remained high.

Posterior deltoid-to-triceps tendon transfer was first described by Moberg^28^ in 1975 using free tendon graft from toe extensors; however, suboptimal functional results were observed with lack of full extension because of elongation of the reconstructed deltoid-triceps muscle-tendon complex. Since then, multiple authors have modified his technique in terms of proximal or distal attachment and types of graft, including a fascia lata autograft; triceps central tendon turn-up with bone block and palmaris longus tendon graft reinforcement; and hamstring, tibialis anterior and synthetic grafts.^2^^,^^29^^,^^30^ The primary advantage of the tendon transfer technique is the well-documented success rates with multiple case series mentioned above, with patients experiencing improved elbow extension and overall function. They are also generally more predictable in terms of outcomes as they do not rely on nerve regeneration, which might vary with patient parameters. Surgeons can also control the tension of transfer intraoperatively and expect similar results postoperatively, and results are immediately seen without a time lag. Wangdell et al^8^ also reported that in addition to the relatively quick restoration of triceps muscle strength after surgery, patients continued to improve beyond the first year of triceps reconstruction, both in terms of performance scores and patient satisfaction. Despite these benefits, tendon transfers require prolonged postoperative immobilization (ie, shoulder abduction brace) or restrictions in shoulder and elbow movements to protect the transferred tendon, as early lengthening of the graft is commonly noted within the first 6 weeks, with an average increase of 2.3 cm.^27^^,^^31^ This poses difficulty in rehabilitation, nursing care, or self-care during the early postoperative period and could potentially lead to stiffness, which complicates rehabilitation. Posterior deltoid-to-triceps tendon transfer is also particularly problematic due to the anatomy of the deltoid muscle. The deltoid muscle lacks a large tendon insertion, and despite including periosteum from the humeral attachment site and the fascia strip from the adjacent brachialis muscle, there is still a large gap of over 15 cm between the deltoid muscle insertion and the triceps aponeurosis, necessitating the use of a tendon graft, which is a common source of complications.^9^ Other surgical complications, such as infection and elbow contracture, were also commonly seen across multiple series, regardless of graft material and transfer techniques.

On the other hand, it is important to bear in mind that biceps-to-triceps tendon transfer is also an option for reconstruction of elbow extension. It is preferred when there is significant flexion contracture of the elbow greater than 45º or weak shoulder stabilizers, such as deltoid and pectoralis major muscles. It is however contraindicated in high cord lesion with nonfunctional biceps, nonfunctional supinator or brachialis muscle, or future planned nerve transfer for restoration of finger and wrist function, such as the commonly performed supinator branch to posterior interosseous nerve transfer. Biceps-to-triceps transfer is technically less demanding with fewer postoperative restrictions. However, postoperative training is more difficult, and there is risk of coactivation of antagonist biceps muscle during elbow extension.^10^^,^^32^ The only randomized controlled trial comparing deltoid-to-triceps transfer and biceps-to-triceps transfer by Mulcahey et al^10^ showed no statistically significant difference between the two groups in terms of elbow extension power; however, the caseload was limited and heterogeneous.

Nerve transfer emerged from the landscape of elbow extension reconstruction in spinal cord injury since the last decade. Bertelli et al^23^ first described transferring the teres minor branch of the axillary nerve to the triceps long head motor branch of the radial nerve in 2011, borrowing the concept of distal nerve transfer in brachial plexus reconstruction. In the subsequent series by Bertelli et al^4^ in 2015, it was reported that the teres minor branch does not always produce strong contractions, so various branches of the axillary nerve, including the posterior division, anterior division and branch to middle deltoid muscle were utilized depending on the presence of strong muscle contraction upon intraoperative nerve stimulation.

Nerve transfers allow early mobilization and rehabilitation and can provide multiple muscle reinnervation with one nerve transfer, especially for those patients with limited tendon transfer options in the lower groups of the International Classification for Surgery of the Hand in Tetraplegia. Despite the theoretical denervation of donor muscles, the nerve transfer was selective because there was no clinically significant weakness in shoulder abduction or external rotation among all reported series. Francoisse et al^33^ attempted to quantify the loss of shoulder abduction strength in their series of nerve transfer surgery in tetraplegia; however, there were limited data (2 limbs with elbow extension reconstruction in 1 patient only). Only a 5.6% decrease in strength at the early time point and a 4.5% decrease at the late time point was reported, which was not significant in daily activities.^33^

The primary limitation of nerve transfer is its inherent unpredictability in comparison to tendon transfers as it is dependent on nerve regeneration. It also has a longer period of lag time before reinnervation is evident, and visible recovery is gradual instead of immediate in tendon transfers. It involves axonal regeneration and reinnervation, central relearning, and a neuroplasticity phase, which takes between 12 and 24 months for most upper limb nerve transfers.^3^ In addition, given that cervical spinal cord injuries can present as a mixture of upper and lower motor neuron lesions, there is still the possibility of muscle denervation and peripheral paralysis. Nerve transfer in tetraplegia therefore still has a time limit compared with pure upper motor neuron lesions, and results would be more promising if performed within 12 months of the index injury.^4^

There are several limitations of this systematic review. First, many of these studies are small case series or case reports, which makes it difficult to draw definitive conclusions. Some studies also only report mean values of manual muscle testing, preventing quantitative evaluation. In addition, variation in surgical techniques that depend on specific patient parameters and the heterogeneous injury pattern in patients with tetraplegia makes direct comparison impossible in this group of patients, and these features represent inherent problems in all studies involving patients with tetraplegia. There is also a lack of long-term follow-up data to determine the durability and long-term outcome of these surgical procedures, especially the newer technique of nerve transfers. Further research is required to determine the true timing of recovery and the sustainability of patient outcomes.

Missing results were observed in multiple studies with potential reporting biases. Although a few studies did not mention the preoperative power, the authors believed that, in general, patients should have a motor power grade of less than 2 to justify performing a tendon transfer in the first place. Thus, all studies demonstrated improvement in motor power after reconstructive surgery, although the effect was minimal in some studies. Publication bias is also likely as surgeons with less favorable outcome and less caseload are less likely to publish their own results.

Moving forward, axillary nerve branch to triceps motor branch transfer is promising, but larger cohorts and case series are required to determine the true complication profile and clinical outcome. Surgeons are encouraged to report their series to produce better quality of collective evidence. At a later stage, larger well-designed trials comparing tendon and nerve transfers in elbow extension reconstruction are necessary to determine the optimal surgical approach in patients with tetraplegia. Additionally, the development of new techniques, modifications, and commercial products to minimize donor nerve morbidity and maximize transfer potentials would further enhance outcomes and patient satisfaction among those undergoing reconstructive surgeries.

In conclusion, reconstruction of elbow extension in patients with tetraplegia provides significant improvement in quality of life, with tendon and nerve transfer representing the primary surgical options. Nerve transfers over the past decade show promising outcomes; however, to date, the evidence is limited to small case series and reports. Although both techniques demonstrate specific advantages and limitations, further research is needed to establish a gold standard treatment and to refine existing approaches for optimal patient outcomes.
